# Mannan-modified adenovirus targeting TERT and VEGFR-2: A universal tumour vaccine

**DOI:** 10.1038/srep11275

**Published:** 2015-06-18

**Authors:** Ying Wang, Jie Zhang, Yang Wu, Zhen-Yu Ding, Xin-Mei Luo, Jie Liu, Wu-Ning Zhong, Guo-Hua Deng, Xiang-Yu Xia, Yao-Tiao Deng, Yu-Quan Wei, Yu Jiang

**Affiliations:** 1 Department of Medical Oncology, Cancer Center, State Key Laboratory of Biotherapy, West China Hospital, Sichuan University, Chengdu 610041, People’s Republic of China; 2 Department of East Ward Oncology, Sichuan Academy of Medical Sciences & Sichuan Provincial People’s Hospital, Chengdu 610072, People’s Republic of China

## Abstract

Antigen-presenting cells including dendritic cells (DCs) express mannan receptors (MR) on their surface, which can be exploited in cancer therapy by designing immune-stimulatory viruses coated with mannan-modified capsids that then bind to DCs and initiate a potent immune response. Although the combination of anti-angiogenesis and cancer immunotherapy agents has a synergistic antitumor effect, more effective strategies for delivering such combinations are still required. Here we report the design and application of mannan-modified adenovirus that expresses both telomerase reverse transcriptase (TERT) and vascular endothelial growth factor receptor-2 (VEGFR-2). Cytotoxic T lymphocytes that are reactive to TERT and VEGFR-2 are capable of mounting an anti-tumour response in murine breast and colon tumour models and in a lung metastatic model. Compared with mannan-modified TERT adenovirus vaccine or mannan-modified VEGFR-2 adenovirus vaccine alone, the combined vaccine showed remarkably synergistic anti-tumour immunity in these models. Both TERT- and VEGFR-2-specific cytotoxic T lymphocytes (CTL) were identified in an *in vitro* cytotoxicity assay, and the CTL activity against tumour cells was significantly elevated in the combined vaccine group. Furthermore, CTL-mediated toxicity was blocked by anti-CD8 monoclonal antibodies. Thus, the combined mannan-modified TERT and VEGFR-2 adenovirus confers potent anti-tumour immunity by targeting both tumour cells and intratumoural angiogenesis.

Tumour progression, growth, and metastasis are intimately associated with both increased intratumoural angiogenesis and increased proliferation. Thus, many therapeutic strategies are focused on targeting either or both of these processes. Angiogenesis, the process by which capillaries sprout from pre-existing blood vessels, is a complex multistep process that is tightly regulated by a large number of angiogenic factors^1^,^2^. Vascular endothelial growth factor (VEGF) and VEGF receptor 2 (VEGFR-2) play pivotal roles in tumour angiogenesis^3^,^4^. VEGFR-2, the major transducer of VEGF-mediated signals that triggers cell proliferation and migration, is extensively expressed in endothelial cells^5^. Inhibition of the VEGF tyrosine kinase signalling pathway inhibits neo-angiogenesis in growing tumours, leading to stasis or regression of tumour growth^6^,^7^,^8^,^9^,^10^,^11^,^12^. This process has clinical relevance, as underscored by the approval of bevacizumab, a humanized anti-VEGF monoclonal antibody, which was the first FDA-approved anti-angiogenic molecular targeting agent. In clinical studies, bevacizumab appears to be more effective when combined with various cytotoxic agents, particularly in the treatment of advanced colorectal cancer and non-small cell lung cancer^13^,^14^,^15^.

Despite this success, anti-angiogenic agents only suppress tumour cell growth and do not elicit complete tumour regression. Indeed, continuous or repeated treatments are required to prevent the re-neovascularization of small residual tumours^16^,^17^. To eradicate the residual tumours, active immunotherapy is a potentially powerful approach^18^,^19^. Moreover, combination of an active immunotherapy that targets tumour cells with anti-angiogenesis drugs can lead to complete tumour regression, as suggested previously^20^. In this context, the exploitation of universal tumour antigens could be used as part of a novel bifunctional anticancer immunotherapy.

Telomerase reverse transcriptase (TERT) is the catalytic subunit of telomerase, which is silent in normal tissues but reactivated in most human and murine cancers. TERT expression is directly correlated with tumour growth and progression^21^,^22^ and TERT serves as a universal tumour antigen in immunotherapy for cancer^23^. Immunization against TERT may overcome tumour cell immune evasion by boosting the level of cytotoxic T cells specifically targeting the neoplasm. Thus, TERT is a promising immunotherapeutic candidate that should be considered for combination with anti-angiogenic therapies.

Co-immunization against tumour (TERT) and angiogenesis-specific markers (VEGFR-2) has a stronger inhibitory effect on tumour growth than single agents^20^. Similarly, immunization of mice with a 1:1 mixture of dendritic cells (DCs) transfected with *VEGFR-2* and *TERT* mRNAs *in vitro* was shown to have a synergistic anti-tumour effect. Nevertheless, preparation of antigen-specific DC vaccine *ex vivo* is costly and time-consuming. A vaccine that directly targets DCs *in vivo* could be used to bypass these high costs and dependency on *ex vivo* manipulation.

Antigen-presenting cells (APCs) including DCs express the mannan receptor (MR) on their surface^24^,^25^. The conjugation of a recombinant antigen and mannan under appropriate oxidized condition can stimulate a measurable antitumor response^26^,^27^. We previously constructed a mannan-modified recombinant TERT adenovirus (AdTERT-m) as a prophylactic vaccine for targeting DCs^28^. This virus stimulated an antigen-specific CTL response against TERT in mice that was correlated with a clear anti-tumour effect. However, in our subsequent study, we found that the therapeutic anti-tumour efficacy of the vaccine was unsatisfactory.

Here, we have dramatically improved the efficacy of this approach by creating a “universal” and effective vaccine, which consists of mannan-modified TERT and VEGFR-2 recombinant adenovirus, Ad (VEGFR2: TERT)-m. The vaccine was tested for its ability to induce anti-tumour immunity in a mouse tumour model. We found that it elicited two kinds of anti-tumour response: an immune cell-mediated attack of tumour cells and suppression of intratumoural angiogenesis.

## Results

### Combination vaccine induced remarkable antitumour effects

**Figure 1:**
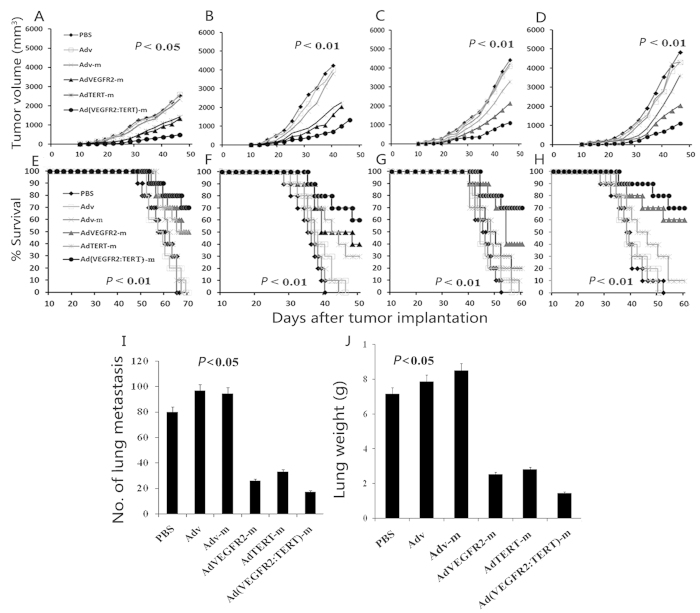
*In vivo* antitumour effects of AdVEGFR2-m and AdTERT-m combination therapy. Mice were immunized with 1 × 10^8^ PFU/100 μl of vaccine once a week for four weeks, and at one week after the fourth immunization they were challenged with 5 × 10^5^ 4T1 (**A** and **E**) and CT26 (**B** and **F**) cells, or they were first challenged subcutaneously with 5 × 10^5^ 4T1 (C and G) and CT26 (D and H) and then after seven days immunized with 1 × 10^8^ PFU/100 μl of vaccine once a week for four weeks. There was a significant difference in tumour size between the combined AdVEGFR2-m and AdTERT-m group and control groups (A-D, *p* < 0.05, repeated measures ANOVA). The combined vaccine Ad(VEGFR2:TERT)-m also prolonged survival of mice compared with controls (E-H, *p* < 0.01, log-rank test). Subsequently, mice were immunized as described above and 5 × 10^5^ LL/2 cells were injected into the tail vein seven days after the last immunization. Once the control mice appeared moribund (21 days after tumour injection), all mice were sacrificed and the resected lungs were weighed and assessed for metastatic nodules on the surface. The combined vaccine decreased the number of metastatic nodules (I) and alleviated tumour burden (J) (*p* < 0.05, one-way variance test). The results were expressed as the mean ± SD.

To determine whether the combination vaccine,Ad (VEGFR2: TERT)-m, induced protective antitumour activity, mice were immunized and then challenged with 4T1 or CT26 tumour cells. As illustrated in Fig. 1, single therapy resulted in a moderate retardation of tumour growth (AdTERT-m or AdVEGFR2-m versus Adv-m, Adv and PBS; *p* < 0.05), and no significant difference was observed in tumour growth inhibition between AdVTERT-m and AdVEGFR2-m. Strikingly, remarkable tumour growth inhibition was achieved after combination vaccine therapy versus AdTERT-m or AdVEGFR2-m alone (*p* < 0.05). In addition, mouse survival was significantly increased in the combination group compared to the other groups (*p* < 0.05). Moreover, anti-tumor effect induced by the vaccines (AdVTERT-m or AdVEGFR2-m alone and the combination vaccine) was lasting for more than 140 days after tumor implantation in CT26 tumor model.

To determine the therapeutic efficiency of the combination therapy in the tumour model, mice were challenged with 5 × 10^5^ live tumour cells in the right flank and one week later received immunization weekly for four continuous weeks. As shown in Fig. 1, tumour growth was slightly retarded in mice in the AdTERT-m group, and there was no significant difference in tumour growth inhibition compared to the Adv-m, Adv and PBS groups. However, significant antitumour activity was observed in the AdVEGFR2-m group in both tumour models. Furthermore, tumour growth in mice treated with the combination vaccine was more significantly inhibited than that in AdTERT-m or AdVEGFR2-m mice (Fig. 1C,D). Synergistic activity of AdVEGFR2-m and AdTERT-m was observed with combination indices under 0.51 (4T1 tumour model) and 0.52 (CT26 tumour model) at a fractional effect of 0.5 (50% tumour cell killing). Thus, AdVEGFR2-m and AdTERT-m acted synergistically, with CI values <0.9 as determined using CalcuSyn Software. Furthermore, survival of the tumour-bearing mice treated with the combination vaccine was much longer than that observed for other groups (Fig. 1G,H).

We also observed that treatment with the combination vaccine in a prophylactic protocol suppressed the formation and growth of lung metastases *in vivo* (Lewis lung carcinoma model). As shown in Fig. 1, the number of surface metastatic nodules was much lower in the combination group than in controls (*p* < 0.05) (Fig. 1I). Additionally, the average lung weight in the combination group was significantly lower than that in the other groups (Fig. 1J).

### Adenovirus immunization increases the number of splenic CD4^+^ T cells and CD8^+^ T cells

**Figure 2:**
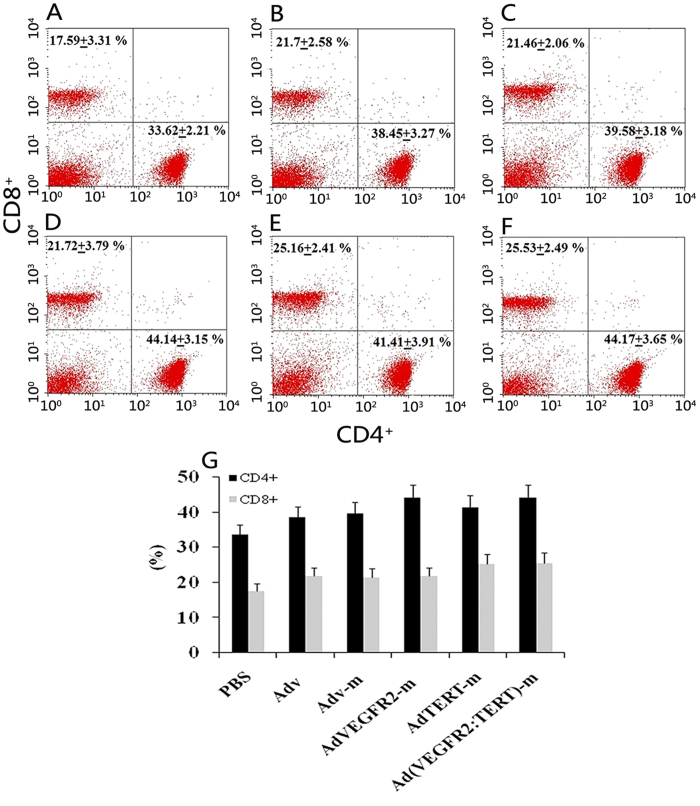
Effect of vaccines on splenic T lymphocytes in tumour-bearing mice. Cell suspensions were derived from spleens of mice immunized over four cycles with PBS (**A**), Adv (**B**), Adv-m (**C**), AdVEGFR2-m (**D**), AdTERT-m (**E**) or Ad(VEGFR2:TERT)-m (**F**). After reaction with mAb to CD4 and CD8, the suspension was subjected to flow cytometry. The proportion of CD8^+^ and CD4^+^ T cells in the vaccinated groups (**B–E**) was markedly higher than that in control (A). Immunotherapy with the AdVEGFR2-m vaccine alone (**D**) increased the number of CD4^+^ T cells (p = 0.006), while the AdTERT-m vaccine alone (**E**) increased the number of CD8^+^ T cells (p = 0.048) compared to the PBS, Adv and Adv-m groups (**A-C**) respectively. Furthermore, immunotherapy with the combination increased both the number of CD4^+^ T cells (p = 0.009) and CD8^+^ T cells (p = 0.034) in the spleen (**F**); (**G**) presents a bar graph.

Following the immunization regimen described above, we analysed the T cell composition in the spleen. As shown in Fig. 2, mice that received immunization with null adenovirus or recombinant adenovirus had a significantly more CD4^+^ T cells and CD8^+^ T cells than mice in the PBS control group (*p* < 0.05). Immunization with the AdVEGFR2-m vaccine alone (Fig. 2D) increased the number of CD4^+^ T cells (p = 0.006), while immunization with the AdTERT-m vaccine alone (Fig. 2E) increased the number of CD8^+^ T cells (p = 0.048) compared with the PBS, Adv and Adv-m groups (Fig. 2A–C). Furthermore, immunization with the combination vaccine increased the number of both CD4^+^ T cells (p = 0.009) and CD8^+^ T cells (p = 0.034) in the spleen (Fig. 2F).

### Mannan-conjugated adenovirus could efficiently target spleen DCs

**Figure 3:**
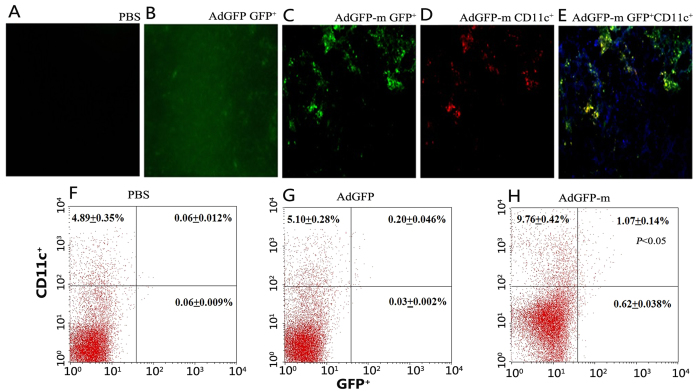
Uptake of adenovirus expressing GFP by DCs in the spleen. Mice were treated i.p. with PBS, AdGFP (B) and AdGFP-m (**C-E**). Three days later, frozen sections of the spleen were stained with a PE-labelled mAb to CD11c and imaged using a fluorescence microscope to determine co-expression of GFP and PE. The PBS group (**A**) was GFP-negative and the AdGFP group (**B**) displayed a non-specific GFP signal. However, cells in the spleen of mice immunized with AdGFP-m displayed both green (**C**) and red (**D**) signals and had a morphology consistent with DCs. Cells expressing GFP colocalized with CD11c (**E**). In parallel, the splenocyte suspensions were prepared and examined by flow cytometry three days after harvesting. The proportion of cells co-expressing GFP and PE in the AdGFP-m group (**H**) was significantly higher than that in the PBS (**F**) and AdGFP group (**G**) (*p* < 0.05, chi-squared test).

To test whether the mannan-conjugated adenovirus could target spleen DCs *in vivo*, mice were immunized with AdGFP-m (i.p.) and the spleens were harvested. As shown in Fig. 3, small clusters of GFP-positive cells were observed in the spleens of mice injected with AdGFP-m (Fig. 3C). Meanwhile,red fluorescence was detected in spleen slices stained for CD11c (Fig. 3D). Moreover, the GFP-expressing cells displayed a micro-morphology consistent with a DC origin and also expressed the DC surface marker CD11c (Fig. 3E). Only non-specific fluorescence was observed in the AdGFP group (Fig. 3B), whereas no fluorescence was detected in the PBS group (Fig. 3A).

To further determine whether the antigen was presented to spleen DCs after vaccination, splenocytes that co-expressed GFP and PE were analysed using flow cytometry. The proportion of cells co-expressing GFP and PE increased significantly in the AdGFP-m group (Fig. 3H) compared with the AdGFP (Fig. 3G) and PBS (Fig. 3F) groups.

### Combination AdTERT-m and AdVEGFR2-m therapy induces greater CTL activity against LL/2 cells than single immunization therapy

**Figure 4:**
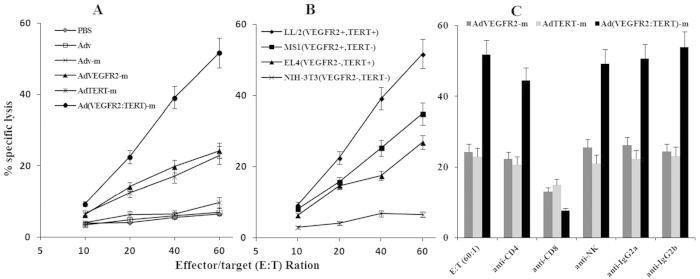
Combination therapy enhanced the cytotoxic effects of CTLs against cancer cells. T cells derived from the spleens of the combined vaccine were tested against LL/2, MS1, EL4 and mouse fibroblast cells (NIH-3T3) at different effector/target ratios using a standard chromium 51Cr release assay. T cells isolated from the spleens of mice treated with the combined vaccine exhibited higher cytotoxicity against LL/2 cells than those from the spleens of controls at a 60:1 effector/target cell ratio (**A**). T cells were able to lyse LL/2, MS1 and EL4 cells (**B**). The cytotoxicity was blocked completely by anti-CD8 (*p* < 0.001, variance test) and partly by anti-CD4 mAbs (**C**). (*p* < 0.05, variance test).

The results showed that the CTLs from mice receiving the single vaccine exhibited enhanced killing activity compared with the control groups and had increased specific lytic activity against LL/2 cells (VEGFR-2^+^ and TERT^+^). The combination vaccine provided the highest CTL activity among all groups, with 51.74 ± 3.35% specific lysis at a 60:1 effector/target ratio (Fig. 4A). There was no specific lytic activity of CTLs against NIH-3T3 cells (VEGFR-2^-^ and TERT^-^) at various target/effector ratios. In addition, the CTL activity against LL/2cells was higher than that against EL4 (VEGFR-2^-^ and TERT^+^) or MS1 (VEGFR-2^+^ and TERT^-^) (Fig. 4B). We also performed a blocking assay at a 60:1 effector/target ratio. Cytotoxicity in the single vaccine group was blocked in the case of the splenocytes pre-treated with anti-CD8 mAb, whereas cytotoxicity in the combination vaccine group was blocked by anti-CD8 or anti-CD4 mAbs (Fig. 4C).

### Enrichment of intra-tumour CD8^+^ T lymphocytes using the combination vaccine

**Figure 5:**
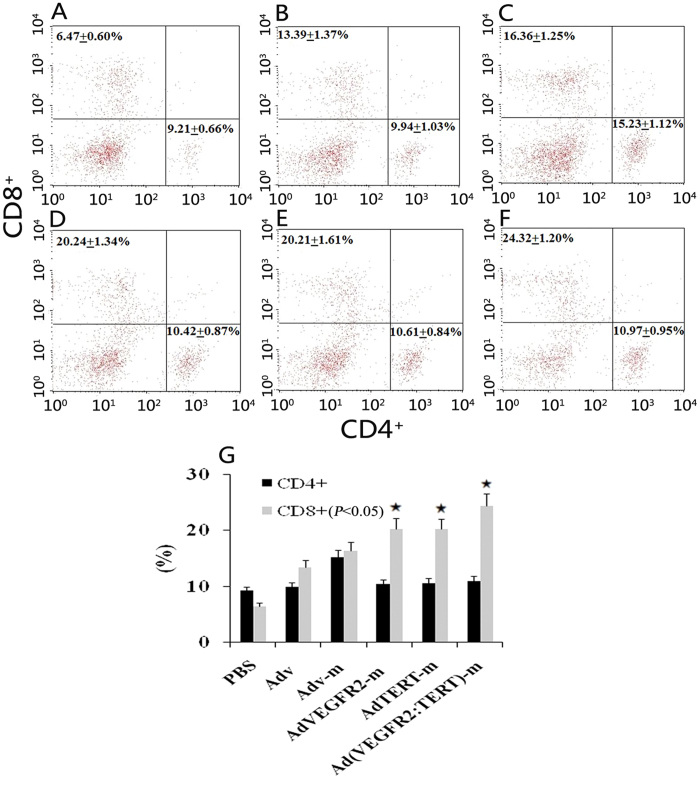
Effect of the combined vaccine on the infiltration of T-cells in tumour tissues. Cell suspensions were prepared from tumour tissues of mice immunized over four cycles with PBS (**A**), Adv (**B**), Adv-m (**C**), AdVEGFR2-m (**D**), AdTERT-m (**E**) or Ad(VEGFR2:TERT)-m (F). After incubation with mAb to CD4 and CD8, the suspension was subjected to flow cytometry. The ratio of CD8^+^ T cells in the combined vaccine group (F) was markedly higher than that observed in the controls (A-E), as shown in the bar graph (G) (*p < 0.05, variance test).

To determine the effect of the combination vaccine on tumour growth and tumour-infiltrating lymphocytes (TIL), we analysed CD4^+^ and CD8^+^ T cell populations. As shown in Fig. 5, separately treatment with AdTERT-m or AdVEGFR2-m increased the CD8^+^ subpopulation of TIL compared to the Adv-m, Adv and PBS groups (*p* < 0.05). However, there was no difference in TIL between AdTERT-m and AdVEGFR2-m. Notably, the proportion of CD8^+^ T-lymphocytes in the combination group was markedly higher than that in the other grou*p*s (*p* < 0.05) (Fig. 5F).

### Immunization with Ad (VEGFR2: TERT)-m induced cytokine secretion

**Figure 6:**
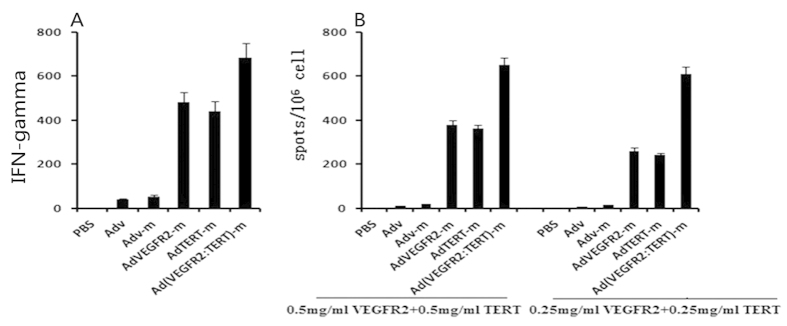
IFN-γ production induced by combined vaccine immunization. The spleens of mice subjected to a complete immunization course were evaluated for IFN-γ production *in vivo* using a mouse IFN-γ ELISA kit (**A**). The amount of IFN-γ in the combined vaccine group was much higher than that in controls. Splenocytes were isolated from mice and incubated on a microplate at 37 °C for 4 h. Two concentrations of proteins (0.5 mg/ml VEGFR-2 with 0.5 mg/ml TERT proteins or 0.25 mg/ml VEGFR-2 with 0.25 mg/ml TERT proteins) were added to each well to stimulate splenocytes. The splenocytes isolated from the combined vaccine group higher levels of IFN-γ than the control groups as determined by the ELISPOT assay (**B**). (*p* < 0.05, variance test).

To quantify the effect of Ad (VEGFR2: TERT)-m vaccination on cytokine secretion, we performed IFN-γ assays for spleen samples of mice that had completed the immunization schedule. The amount of IFN-γ in the spleens of the Ad (VEGFR2: TERT)-m group was distinctly higher than that in the spleens of the other groups (Fig. 6A). A similar result was observed in the ELISPOT assay (Fig. 6B).

### Inhibition of angiogenesis following mannan-modified adenovirus immunization

**Figure 7:**
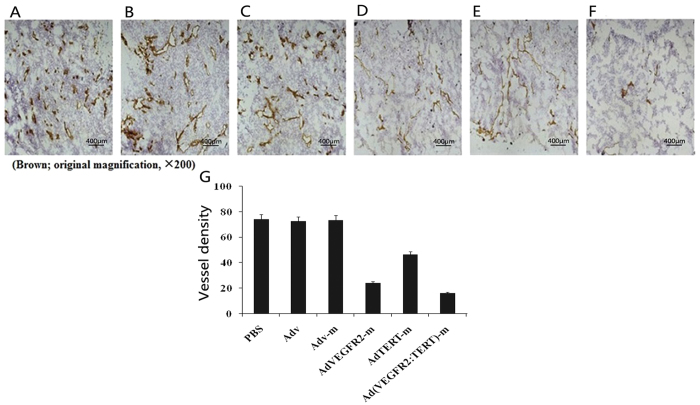
Anti-angiogenesis effect of combined vaccine immunization. Frozen sections of B16 tumour tissues isolated from mice immunized with PBS (**A**), Adv (**B**), Adv-m (**C**), AdVEGFR2-m (**D**), AdTERT-m (**E**) and Ad(VEGFR2:TERT)-m (**F**) were stained with an antibody reactive to CD31. The vessel density of each sample was estimated by averaging the total number of microvessels in five random fields (brown; original magnification, ×200). The vessel density in the Ad (VEGFR2:TERT)-m group was significantly lower than that in the other groups (**G**) (*p* < 0.05, variance test).

Next, the anti-angiogenic effect of the vaccines was explored. As shown in Fig. 7, tumour angiogenesis in the AdVEGFR2-m group was clearly suppressed compared with the control group (Fig. 7A–C), and this effect was markedly enhanced upon combination with the AdTERT-m virus (Fig. 7A–E). A significant reduction in microvessel density was also observed (Fig. 7G).

### Combined immunotherapy is not overtly toxic to normal tissues

Vaccinated animals with no evidence of tumours were evaluated for potential toxicity for more than 10 months. No adverse consequences were indicated by gross measures including weight loss, ruffling of fur, life span, behaviour or feeding. No pathologic changes were evident in the liver, lung, kidney, spleen, or heart upon microscopic examination.

To examine the effects of immunotherapy on fecundity, female mice were immunized with combined Ad (VEGFR2: TERT)-m or each individual virus once a week for four continuous weeks and were then allowed to cohabitate with non-immunized males. Under our experimental conditions, none of the females in any of the experimental groups became pregnant.

## Discussion

This study demonstrated that combination immunotherapy was effective and simple to deliver. Previous studies have shown that mannan modification significantly enhances antigen identification and uptake by splenic DCs^28^, or the lymph nodes DCs^29^, and leads to the activation of specific T lymphocytes. It was shown that spleen DCs could process and present antigens efficiently irrespective of the route of antigen capture^30^, therefore we take the convenience to manipulate and more intuitive method (spleen sections) to investigate the antigens delivering into DCs. Consistent with these observations, our mannan-modified vaccine is able to deliver tumour antigens to DCs and then stimulate an anti-tumour immune response (Fig. 3). Compared with previous single antigen-encoding strategies developed in our laboratory^28^, the combination virus was more effective for stimulation of DCs and provided a more potent anti-tumour immune response that significantly improved survival.

A subcutaneous tumour model was conducted in our present work to establish an initial assessment of efficacy of the vaccine *in vivo*^31^,^32^,^33^,^34^. The remarkable synergistic effect on tumour inhibition and mouse survival prolongation when AdTERT-m and AdVEGFR2-m were combined indicates that integration of anti-angiogenesis and antitumour immunity into one immunotherapy is a promising approach. This is clinically relevant because VEGFR-2 is also expressed by immunosuppressive cells. Thus, blockade of the VEGF-VEGFR-2 pathway could inhibit tumour-induced regulatory T cell proliferation and thereby further alleviate immunosuppression in the tumour microenvironment^35^,^36^. Our data also indicate that the ability of the combined vaccine to suppress angiogenesis is an effective strategy to enhance immunotherapy. The results of present work provide an initial assessment of vaccine’s efficacy for further studies, such as studies with orthotopic tumour models or in large animals or humans. Our approach thus provides the experimental and conceptual basis for the design of novel future clinical strategies.

Recombinant viral vaccines are commonly studied for anticancer vaccination. In practice, however, the neutralizing immune response against viral proteins reduces the efficacy of this approach during repeated immunizations. However, replication-deficient adenoviruses can be amplified to high titres and used to stimulate the production of abundant CD8^+^ T cells^37^. Adenoviruses are also efficient vehicles that can be used to elicit long-term immunological memory and immune responses^38^, which is especially suitable for the purposes of the current study. We previously showed that AdTERT-m treatment did not inhibit tumour growth to the extent that we predicted^28^. Our finding that AdVEGFR2-m immunotherapy was superior to that of AdTERT-m *in vivo* (Fig. 1C,D) is consistent with the results of Nair *et al.*, who reported that anti-TERT immunotherapy was inferior to anti-VEGF/VEGFR-2 treatment^20^. This indicates that anti-VEGFR-2 treatment has inhibitory effects on both tumour vasculature and tumour cells themselves.

We suggested that the combined vaccine was clearly superior to either of the single vaccines because of a potential convergence on the CTL response, as previous studies have shown that this is the primary mechanism responsible for tumour regression in mice^28^,^29^. Indeed, we found that combined vaccine treatment induced cytotoxicity more robustly in tumour cells expressing either VEGFR-2 or TERT (MS1 and EL4 cells, respectively) and was even more effective against LL/2 cells, which express both antigens (Fig. 4B). Further analysis revealed that the effect of the combined vaccine was almost completely attenuated by the depletion of CD8^+^ T lymphocytes (Fig. 4C). These findings indicated that the selective CTL response against TERT and VEGFR-2 induced by the combined vaccine may rely mainly on CD8^+^ T lymphocytes. This is consistent with the critical role of CD8^+^ T cells in the development and maintenance of CTLs and in mediating antitumour immunity^39^,^40^,^41^,^42^.

Increased intra-tumour infiltration of lymphocytes (TILs) and activation of CD8^+^ T-cells^43^ inhibits tumour growth *in vivo*^44^. Moreover, CD8^+^ T-cell infiltration in colon cancer^45^, breast cancer^46^ and lung cancer^47^ is the best prognostic marker for patient survival. In addition, CD8^+^ T cells are the dominant element of TILs^47^, and CD8^+^ T cell or TIL function is always impaired in tumours^48^. On the basis of these findings, we measured CD8^+^ T cell abundance in tumour tissues and found that the intra-tumour infiltration of CD8^+^ T lymphocytes in the combined vaccine group was significantly increased (Fig. 5). Thus, the higher proportion of CD8^+^ T lymphocytes may be at least partly responsible for the antitumour efficacy of our combined adenoviral therapy.

We note, however, that the effects of the combined vaccine were also partially inhibited by depletion of CD4^+^ T lymphocytes (Fig. 4C). Thus, CD4^+^ T lymphocytes may also mediate a specific CTL response against TERT and VEGFR-2^49^. This is consistent with observations that IFN-γ secreted by CD4^+^ T lymphocytes plays an important role in anti-tumour immunity^50^, and that those CD4^+^ T lymphocytes regulate early tumour outgrowth via IFN-γ-dependent inhibition of tumour angiogenesis^51^. Furthermore, CD4^+^ T cells promote the activation and proliferation of CD8^+^ T lymphocytes in advanced tumours^52^,^53^. In our current study, we observed that the combined vaccine leaded to the generation of high levels of IFN-γ (Fig. 6). Together, these results suggested that both CD8^+^ and CD4^+^ T lymphocytes are involved in the immune response against TERT and VEGFR-2.

We also observed inhibition of angiogenesis in mouse tumour tissue (Fig. 7), consistent with the reduced rate of angiogenesis in mice immunized against VEGFR-2. In the combined vaccine group, an extraordinary suppression of tumour-associated angiogenesis was observed, confirming that immunization against TERT and VEGFR-2 is a powerful anti-angiogenic strategy. The mechanisms underlying this synergy are not well understood but may involve a reduction in the secretion of pro-angiogenic factors from the tumour cells themselves.

Although no significant impact on overall physiology was detected in males or females over the long term in response to the combined virus, we did find that the therapy was associated with an abolition of fecundity. This is consistent with a previous report that mice immunized against VEGFR-2 fail to become pregnant^54^. The contribution of TERT immunization to this effect is not clear, although Nair *et al.* did not detect an impact of TERT on pregnancy^20^. Thus, the potential adverse effects of immunotherapy and their underlying causes should be carefully considered in future experiments.

To the best of our knowledge, we believe that the present report provides the first description of a combined tumour immunotherapy that targets both TERT and VEGFR-2. The reduced requirement for *ex vivo* manipulation and the remarkable synergy achieved by targeting both tumour cells and tumour vasculature suggest that this approach is may be suitable for translation to future clinical studies.

## Materials and Methods

### Ethics statement

Mouse experiments were conducted in accordance with Sichuan University’s animal welfare guidelines. The experiments were approved by the the Ethics Committee of Animal Experimentation of Sichuan University.

### Adenovirus construction

Recombinant adenoviruses expressing TERT and VEGFR-2 were constructed and identified in preliminary work^28^,^55^. Briefly, murine VEGFR-2 and TERT were amplified from the VEGFR-2 and TERT original plasmids using published primers. Using the SfiI restriction enzyme (New England Biolabs) and T4 DNA ligase (JingMei Biological Engineering Co., Ltd), the target genes were subsequently cloned into a shuttle vector (pShuttle-CMV, SinoGenoMox Research Center Co., Ltd). The recombinant vectors (pShuttle-VEGFR2 and pShuttle-TERT) were transferred into the adenoviral backbone carrier pAdxsi (SinoGenoMoxResearch Center Co., Ltd) using I-CeuI and I-SceI restriction enzymes (New England Biolabs) and T4 DNA ligase (JingMei Biological Engineering Co., Ltd). pAdxsi-VEGFR2 and pAdxsi-TERT were linearized using PacI restriction enzyme (New England Biolabs) and transfected into HEK293 cells (human embryonic kidney cell line). After 14 days, adenoviruses (AdVEGFR-2 and AdTERT) were harvested. The recombinant adenoviruses were amplified in HEK293 cells, purified with the Adenovirus Purification Miniprep Kit (Biomiga San Diego, USA), and further titred by using a plaque assay, yielding the number of plaque forming units per millilitre (PFU/ml). To investigate the process of antigen uptake, an adenovirus vector expressing green fluorescent protein (AdGFP) was also prepared.

### Adenovirus modification

The recombinant adenovirus was coupled with mannan (Sigma) as previously reported^56^. In brief, adenoviruses were mixed with oxidizing mannan (OM) at a proportion of 1 × 10^8^ PFU:14 mg/ml and incubated overnight at room temperature (RT) for AdTERT-m (AdTERT-mannan) or AdVEGFR2-m (AdVEGFR2-mannan) generation without any further purification. We observed that co-immunization of mice with dendritic cells (DCs) transfected with mRNAs that encode TERT and VEGFR-2 (mixed at a 1:1 ratio) exhibited a synergistic antitumour effect, as reported previously^20^. We thus used recombinant adenovirus AdTERT-m and AdVEGFR2-m mixed at the ratio of 1:1 (1 × 10^8^ PFU: 1 × 10^8^ PFU) as our combination vaccine Ad(VEGFR2:TERT)-m (Ad(VEGFR2:TERT)-mannan). Additionally, we used phosphate-buffered saline (PBS), Ad-null virus (without the genes of interest) modified with or without OM (Adv-m and Adv, respectively), AdTERT-m alone and AdVEGFR2-m alone as controls.

### Tumour models and immunization

Female 6-week-old mice were purchased from the Laboratory Animal Center of Sichuan University. To investigate the preventive effect of Ad(VEGFR2:TERT)-m in tumour models, mice were randomly divided into 6 groups (n = 10 animals/group) and a dose of 1 × 10^8^ PFU/100 μl per mouse of Ad(VEGFR2:TERT)-m (the dose proved effective in preliminary experiments) was delivered to the mice by intraperitoneal (i.p.) injection once a week for four weeks. Control animals were given i.p. injections of AdTERT-m alone, AdVEGFR2-m alone, Adv, Ad-m or PBS. One week after the fourth immunization, 4T1 murine mammary tumour cells or CT26 murine colon carcinoma cells (5 × 10^5^) were injected subcutaneously into the right flanks of BALB/c mice. In the therapeutic immunization mode, mice (n = 10 per group) were injected with 5 × 10^5^ live tumour cells subcutaneously in the flank. Seven days later, mice were given i.p. injections of the vaccines weekly for four weeks at the same dose as described above. Tumour incidence was evaluated by daily inspection and tumour volume was measured every 3 days using a Vernier caliper. Volume was calculated according to the following formula: V = 0.52 × L (W) ^2^. V = volume (mm^3^), L = length (mm) and W = width (mm)^57^.

To test the efficacy of vaccines against tumour metastasis, 5 × 10^5^ LL/2 (Lewis lung carcinoma) cells were injected into the tail vein of each C57BL/6 mouse on day 7 after the fourth immunization (as described above). All mice were sacrificed when control mice became moribund (21 days after LL/2 cells were injected into the tail vein of each mouse). Lung surface metastatic nodules and lung weight were measured^58^.

### Targeting splenic dendritic cells (DCs) using adenovirus modified with oxidized mannan

To investigate the process of antigen uptake, BALB/c mice (n = 5 per group) were injected i.p. with 1 × 10^8^ PFU of oxidized mannan-modified AdGFP (AdGFP-m), unmodified AdGFP or PBS. Three days later, the spleens were removed from recipient mice. One half of the spleen was cryosectioned and sections were stained with phycoerythrin (PE)-labelled anti-CD11c (BD Pharmingen). The two-color immunofluorescence (PE and GFP) was examined using fluorescence microscopy (Zeiss). The other half of the spleen was prepared for single splenocyte suspension and stained with PE-labelled anti-CD11c for analysis by flow cytometry (Becton Dickinson, San Jose, USA).

### *In vitro* cytotoxicity assay

Single-cell splenocyte suspensions were prepared from recipient mice one week after immunization and used as cytotoxic T-lymphocyte (CTL) effector cells. MS1 (a murine pancreatic endothelial cell line expressing VEGFR-2), EL4 (a VEGFR-2-negative murine lymphoma cell line), LL/2 (a Lewis lung carcinoma cell line expressing both VEGFR-2 and TERT) and NIH/3T3 (a murine embryonic fibroblast cell line expressing neither VEGFR-2 nor TERT) cells were used as target cells. Effector and target cells were incubated at different ratios in 96-well plates. The cytolytic activity of the cells was determined using a 4-h chromium ^51^Cr release assay. Briefly, approximately 1 × 10^6^ target cells were labelled with 100 μCi of Na_2_^51^CrO4 (Amersham Bioscience) for 2 h at 37 °C and then plated at a concentration of 1 × 10^4^/100 μl/well into 96-well plates. Next, 100 μl of splenocytes was added at different ratios to ^51^Cr-labeled target cells and incubated for 4 h at 37 °C. Target cells cultured in medium alone (target spontaneous release) or treated with 1% Triton X-100 (target maximum release) were used as controls. Finally, 100 μl of the cell supernatant was harvested and evaluated for radioactivity using a gamma counter. Cytotoxicity was calculated using the following formula: lysis rate (%) = 100% × [(experimental release - target spontaneous release)/(target maximum release - target spontaneous release)].

In addition, cytotoxicity inhibition assays were also performed. Target cells were pre-treated with monoclonal antibodies (mAbs) (BD Pharmingen) at 50 mg/ml at room temperature for 30 min. The mAbs used included anti-CD4, anti-CD8, anti-NK, and their isotype-matched IgG controls. We validated the activity of each of these mAbs in preliminary pilot experiments.

### ELISA

Seven days after the fourth immunization, splenic mononuclear cells were isolated from individual mice, and the splenocytes (2 × 10^5^/well) were cultured in 96-well culture plates at 37 °C with 5% CO_2_ in the presence or absence of VEGFR-2 and TERT proteins (0.5 mg/ml, respectively). The supernatants were collected at 72 h to analyse the secretion of IFN-γ using an enzyme-linked immunosorbent assay ELISA kit (Neobioscience Technology Co., Ltd).

### ELISPOT assay

The enzyme-linked immunospot (ELISPOT) assay was performed to assess IFN-γ secretion. Briefly, splenocytes (2 × 10^5^/well) were incubated in polyvinylidene difluoride (PVDF)–bottom 96-well filtration plates (Dakewe Biotech Company Limited) at 37 °C with 5% CO_2_ in the presence or absence of VEGFR-2 and TERT proteins (BD Pharmingen) (0.5 mg/ml VEGFR-2 with 0.5 mg/ml TERT or 0.25 mg/ml VEGFR-2 with 0.25 mg/ml TERT) for 4 h. Once IFN-γ was produced, wells were read using an ELISPOT reader (Cellular Technology Ltd.). The numbers of spot-forming cells (SFC) per 10^6^ cells was calculated.

### Detection of CD4^+^ and CD8^+^ T cells via flow cytometry

Single-cell splenocyte suspensions were prepared from recipient mice seven days after immunization as mentioned above. Cells were stained with anti-CD8-FITC, anti-CD4-PE, or isotype control (BD Pharmingen). The frequency of CD4^+^ and CD8^+^ T cells was characterized using flow cytometry analysis on a FACScan system with CellQuest software (BD Pharmingen).

In addition, the amount of lymphocytes that infiltrated tumour issues was evaluated. Mice were challenged with LL/2 cells (5 × 10^5^ cells per mouse). One week after four cycles of immunization, tumour tissues were removed and suspensions of individual tumour cells were prepared by straining samples through 80-μm mesh nylon wool. After addition of ammonium chloride lysis buffer, cells were incubated with anti-CD4-PE or anti-CD8-FITC and then analysed using flow cytometry as described above.

### Immunohistochemistry

Immunohistochemistry was performed as described previously^59^. To investigate the microvessel density (MVD) in tumour tissues, frozen sections were fixed in acetone and stained with monoclonal rat anti-mouse CD31 (murine endothelial cell marker, BD Pharmingen). The MVD was estimated by averaging the total number of microvessels from five high-power fields per section as described previously^60^.

### Evaluation of adverse effects

Mice immunized with these vaccines were also investigated for potential toxic side effects of the immunization. The evaluation continued for over 10 months. Gross measures such as weight loss, ruffling of fur, life span, behaviour, and feeding were investigated. Tissues of the heart, liver, spleen, lung, and kidney were also fixed in 10% neutral buffered formalin solution and embedded in paraffin. Sections with a thickness of 3–5 μm were stained with haematoxylin and eosin (H&E).

Fertility was tested as reported previously^60^. Briefly, mice were immunized with Ad(VEGFR2:TERT)-m, AdTERT-m or AdVEGFR2-m alone four times at weekly intervals, and female mice were then allowed to cohabitate with non-immunized males. This was performed in triads (two females to one male per cage). The number of days until labour and the number of pups were recorded.

### Statistical analyses

All data are presented as the mean ± standard deviation (SD) and are representative of at least triplicate experiments. The differences between individual time points and differences between the groups were analysed using ANOVA and unpaired Student’s t test. The survival curves were plotted using the Kaplan-Meier method and analysed between groups using the standard Mantel-Cox log-rank test. Ratios were compared using the chi-squared test. Significant differences were considered at *p* < 0.05. The nature of the interaction between AdTERT-m and AdVEGFR2-m was calculated using the Chou-Talalay method for determining the combination index (CI) using CalcuSyn software (Biosoft, Ferguson, MO). Based on this approach, combination index values <0.9 are considered synergistic, values >1.1 are antagonistic, and values 0.9–1.1 are nearly additive^61^.

## Additional Information

**How to cite this article**: Wang, Y. *et al.* Mannan modified adenovirus targeting TERT and VEGFR-2: A universal tumor vaccine. *Sci. Rep.* **5**, 11275; doi: 10.1038/srep11275 (2015).

## References

[b1] Risau W. Mechanisms of angiogenesis. Nature. 386, 671–4 (1997). 9109485 10.1038/386671a0

[b2] Gaspariru G. The rationale and future potential of angiogenesis inhibitors in neoplasia. Drugs. 58, 17–38 (1999). 10.2165/00003495-199958010-00003 10439927

[b3] Vajkoczy P. *et al.* Microtumor growth initiates angiogenic sprouting with simultaneous expression of VEGF, VEGF receptor-2, and angiopoietin-2. J Clin Invest. 109, 777–85 (2002). 11901186 10.1172/JCI14105 PMC150910

[b4] McMahon G. VEGF receptor signaling in tumor angiogenesis. Oncologis. 5, 3–10 (2000). 10.1634/theoncologist.5-suppl_1-3 10804084

[b5] Gille H. *et al.* Analysis of biological effects and signaling properties of Flt-1 (VEGFR-1) and KDR (VEGFR-2). J Biol Chem 276, 3222–30 (2001). 11058584 10.1074/jbc.M002016200

[b6] Wang B., Kaumaya P. T. & Cohn D. E. Immunization with synthetic VEGF peptides in ovarian cancer. Gynecol Oncol. 119, 564–70 (2010). 20822802 10.1016/j.ygyno.2010.07.037 PMC4289616

[b7] Morera Y. *et al.* Anti-tumoral effect of active immunotherapy in C57BL/6 mice using a recombinant human VEGF protein as antigen and three chemically unrelated adjuvants. Angiogenesis. 11, 381–93 (2008). 19034678 10.1007/s10456-008-9121-5

[b8] Dong J. *et al.* A comparative study of gene vaccines encoding different extracellular domains of the vascular endothelial growth factor receptor 2 in the mouse model of colon adenocarcinoma CT-26. Cancer Biol Ther. 7, 502–9 (2008). 18285704 10.4161/cbt.7.4.5477

[b9] Zhang H. *et al.* Antiangiogenic immunotherapy targeting Flk-1, DNA vaccine and adoptive T cell transfer, inhibits ocular neovascularization. Biochem Biophys Res Commun. 381, 471–476 (2009). 19340941 10.1016/j.bbrc.2009.01.178

[b10] Bequet-Romero M. *et al.* Prophylactic naked DNA vaccination with the human vascular endothelial growth factor induces an anti-tumor response in C57Bl/6 mice. Angiogenesis. 10, 23–34 (2007). 17273909 10.1007/s10456-006-9062-9

[b11] Wei Y. *et al.* Enhancement of DNA vaccine efficacy by targeting the xenogeneic human chorionic gonadotropin, survivin and vascular endothelial growth factor receptor 2 combined tumor antigen to the major histocompatibility complex class II pathway. J Gene Med. 14, 353–62 (2012). 22438278 10.1002/jgm.2624

[b12] Luo Y., Markowitz D., Xiang R., Zhou H. & Reisfeld R. A. FLK-1-based minigene vaccines induce T cell-mediated suppression of angiogenesis and tumor protective immunity in syngeneic BALB/c mice. Vaccine. 25, 1409–15 (2007). 17113202 10.1016/j.vaccine.2006.10.043 PMC1995657

[b13] Hurwitz H. *et al.* Bevacizumab plus irinotecan, fluorouracil, and leucovorin for metastatic colorectal cancer. N Engl J Med. 350, 2335–42 (2004). 15175435 10.1056/NEJMoa032691

[b14] Escudier B. *et al.* Bevacizumab plus interferon alfa-2a for treatment of metastatic renal cell carcinoma:a randomised, double-blind phase III trial. Lancet. 370, 2103–11 (2007). 18156031 10.1016/S0140-6736(07)61904-7

[b15] Sandler A. *et al.* Paclitaxel-carboplatin alone or with bevacizumab for non-small-cell lung cancer. N *Engl J Med*. 355, 2542–50 (2006). 17167137 10.1056/NEJMoa061884

[b16] Ma J. & Waxman D. J. Dominant effect of antiangiogenesis in combination therapy involving cyclophosphamide and axitinib. Clin Cancer Res. 15, 578–88 (2009). 19147763 10.1158/1078-0432.CCR-08-1174 PMC2729124

[b17] Blagosklonny M. V. Antiangiogenic therapy and tumor progression. Cancer Cell 5, 13–7 (2004). 14749122 10.1016/s1535-6108(03)00336-2

[b18] Hall S. S. A Commotion in the Blood: Life, Death, and the Immune System. [Hall S. S. (ed.)] (New York, 1997).

[b19] Sylvester R. J. Bacillus Calmette-Guerin treatment of non-muscle invasive bladder cancer. Int J Urol. 18, 113–20 (2011). 21091799 10.1111/j.1442-2042.2010.02678.x

[b20] Nair S. *et al.* Synergy between tumor immunotherapy and antiangiogenic therapy. Blood 102, 964–71 (2003). 12689940 10.1182/blood-2002-12-3738

[b21] Kim N. W. *et al.* Specific association of human telomerase activity with immortal cells and cancer. Science. 266, 2011–5 (1994). 7605428 10.1126/science.7605428

[b22] Meyerson M. *et al.* hEST, the putative human telomerase catalytic subunit gene, is up-regulated in tumor cells and during immortalization. Cell. 90, 785–95 (1997). 9288757 10.1016/s0092-8674(00)80538-3

[b23] Vonderheide R. H. Telomerase as a universal tumor-associated antigen for cancer immunotherapy. Oncogene. 21, 674–679 (2002). 11850795 10.1038/sj.onc.1205074

[b24] East L. & Isacke C. M. The mannose receptor family. Biochim Biophys Acta. 1572, 364–86 (2002). 12223280 10.1016/s0304-4165(02)00319-7

[b25] Irjala H. *et al.* Mannose receptor is a novel ligand for L-selectin and mediates lymphocyte binding to lymphatic endothelium. J Exp Med. 194, 1033–42 (2001). 11602634 10.1084/jem.194.8.1033 PMC2193520

[b26] Condaminet B. *et al.* Human epidermal Langerhans cells express the mannose–fucose binding receptor. Eur J Immunol. 28, 3541–51 (1998). 9842897 10.1002/(SICI)1521-4141(199811)28:11<3541::AID-IMMU3541>3.0.CO;2-4

[b27] Apostolopoulos V., Pietersz G. A., Loveland B. E., Sandrin M. S. & McKenzie I. F. Oxidative/reductive conjugation of mannan to antigen selects for T1 or T2 immune responses. Proc Natl Acad Sci U S A. 92, 10128–32 (1995). 7479739 10.1073/pnas.92.22.10128 PMC40749

[b28] Ding Z. *et al.* Mannan-modified adenovirus as a vaccine to induce antitumor immunity. Gene Ther. 14, 657–63 (2007). 17287861 10.1038/sj.gt.3302893

[b29] Zhao Z. *et al.* Antitumour immunity mediated by mannan-modified adenovirus vectors expressing VE-cadherin. Vaccine 29, 4218–24(2011). 21497630 10.1016/j.vaccine.2011.03.109

[b30] Kamphorst A. O. *et al.* Route of antigen uptake differentially impacts presentation by dendritic cells and activated monocytes. J Immunol. 185, 3426–35 (2010). 20729332 10.4049/jimmunol.1001205 PMC3013633

[b31] Céspedes M. V., Casanova I., Parreño M. & Mangues R. Mouse models in oncogenesis and cancer therapy. Clin Transl Oncol. 8, 318–29 (2006). 16760006 10.1007/s12094-006-0177-7

[b32] Ruggeri B. A., Camp F. & Miknyoczki S. Animal models of disease: pre-clinical animal models of cancer and their applications and utility in drug discovery. Biochem Pharmacol. 87, 150–61 (2014). 23817077 10.1016/j.bcp.2013.06.020

[b33] Jung J. Human tumor xenograft models for preclinical assessment of anticancer drug development. Toxicol Res. 30, 1–5 (2014). 24795792 10.5487/TR.2014.30.1.001 PMC4007037

[b34] Richmond A. & Su Y. Mouse xenograft models vs GEM models for human cancer therapeutics. Dis Model Mech. 1, 78–82 (2008). 19048064 10.1242/dmm.000976 PMC2562196

[b35] Terme M. *et al.* VEGFA-VEGFR pathway blockade inhibits tumor-induced regulatory T-cell proliferation in colorectal cancer. Cancer Res. 73, 539–49 (2013). 23108136 10.1158/0008-5472.CAN-12-2325

[b36] Tartour E. *et al.* Angiogenesis and immunity: a bidirectional link potentially relevant for the monitoring of antiangiogenic therapy and the development of novel therapeutic combination with immunotherapy. Cancer Metastasis Rev. 30, 83–95 (2011). 21249423 10.1007/s10555-011-9281-4

[b37] Kochenderfer J. N. & Gress R. E. A comparison and critical analysis of preclinical anticancer vaccination strategies. Exp Biol Med (Maywood). 232, 1130–41 (2007). 17895521 10.3181/0702-MR-42

[b38] Murali-Krishna K. *et al.* Counting antigen-specific CD8 T cells: a reevaluation of bystander activation during viral infection. Immunity. 8, 177–87 (1998). 9491999 10.1016/s1074-7613(00)80470-7

[b39] Gattinoni L. *et al.* CTLA-4 dysregulation of self/tumor-reactive CD8+ T-cell function is CD4+ T-cell dependent. Blood. 108, 3818–23 (2006). 16882704 10.1182/blood-2006-07-034066 PMC1679662

[b40] Wang R. F. The role of MHC class II-restricted tumor antigens and CD4+ T cells in antitumor immunity. Trends Immunol. 22, 269–76 (2001). 11323286 10.1016/s1471-4906(01)01896-8

[b41] Shedlock D. J. & Shen H. Requirement for CD4 T cell help in generating functional CD8 T cell memory. Science. 300, 337–9 (2003). 12690201 10.1126/science.1082305

[b42] Ostrand-Rosenberg S. CD4+ T lymphocytes: a critical component ofantitumor immunity. Cancer Invest. 23, 413–9 (2005). 16193641

[b43] Lau B. H., Yamasaki T. & Gridley D. S. Garlic compounds modulate macrophage and T-lymphocyte functions. Mol Biother. 3, 103–7 (1991). 1910619

[b44] Ebrahimi M., Mohammad Hassan Z., Mostafaie A., Zare Mehrjardi N. & Ghazanfari T. Purif ied Protein Fraction of Garlic Extract Modulates Cellular Immune Response against Breast Transplanted Tumors in BALB/c Mice Model. Cell J. 15, 65–75 (2013). 23700562 PMC3660026

[b45] Galon J. *et al.* Type, density, and location of immune cells within human colorectal tumors predict clinical outcome. Science. 313, 1960–4 (2006). 17008531 10.1126/science.1129139

[b46] Seo A. N. *et al.* Tumour-infiltrating CD8+ lymphocytes as an independent predictive factor for pathological complete response to primary systemic therapy in breast cancer. Br J Cancer. 109, 2705–13 (2013). 24129232 10.1038/bjc.2013.634 PMC3833219

[b47] Prado-Garcia H., Romero-Garcia S., Aguilar-Cazares D., Meneses-Flores M. & Lopez-Gonzalez J. S. Tumor-induced CD8+ T-cell dysfunction in lung cancer patients. Clin Dev Immunol 2012, 741741 (2012). 23118782 10.1155/2012/741741 PMC3483679

[b48] Gajewski T. F. *et al.* Cancer immunotherapy strategies based on overcoming barriers within the tumor microenvironment. Curr Opin Immunol. 25, 268–276 (2013). 23579075 10.1016/j.coi.2013.02.009

[b49] Cheroutre H. & Husain M. M. CD4 CTL: Living up to the challenge. Semin Immunol. 25, 273–81 (2013). 24246226 10.1016/j.smim.2013.10.022 PMC3886800

[b50] Qin Z. *et al.* A critical requirement of interferon gamma-mediated angiostasis for tumor rejection by CD8+ T cells. Cancer Res. 63, 4095–100 (2003). 12874012

[b51] Beatty G. & Paterson Y. IFN-gamma-dependent inhibition of tumor angiogenesis by tumor-infiltrating CD4+ T cells requires tumor responsiveness to IFN-gamma. J Immunol. 166, 2276–82 (2001). 11160282 10.4049/jimmunol.166.4.2276

[b52] Schultz R. M. & Kleinschmidt W. J. Functional identity between murine gamma interferon and macrophage activating factor. Nature. 305, 239–40 (1983). 6412144 10.1038/305239a0

[b53] Pace J. L., Russell S. W., Torres B. A., Johnson H. M. & Gray P. W. Recombinant mouse gamma interferon induces the priming step in macrophage activation for tumor cell killing. J Immunol. 130, 2011–3 (1983). 6403616

[b54] Zhang L. *et al.* Intratumoral T cells, recurrence, and survival in epithelial ovarian cancer. N Engl J Med. 348, 203–13 (2003). 12529460 10.1056/NEJMoa020177

[b55] Zhang J. *et al.* Mannan-modified adenovirus encoding VEGFR-2 as a vaccine to induce anti-tumor immunity. J Cancer Res Clin Oncol 140, 701–12 (2014). 24525706 10.1007/s00432-014-1606-6 PMC11823742

[b56] Stambas J., Pietersz G., McKenzie I. & Cheers C. Oxidized mannan as a novel adjuvant inducing mucosal IgA production. Vaccine. 20, 1068–78 (2002). 11803067 10.1016/s0264-410x(01)00456-x

[b57] Sauter B. V., Martinet O., Zhang W. J., Mandeli J. & Woo S. L. Adenovirus-mediated gene transfer of endostatin *in vivo* results in a high level of transgene expression and inhibition of tumor growth and metastases. Proc Natl Acad Sci U S A. 97, 4802–7 (2000). 10758166 10.1073/pnas.090065597 PMC18313

[b58] Lozonschi L. *et al.* Controlling tumor angiogenesis and metastasis of C26 murine colon adenocarcinoma by a new matrix metalloproteinase inhibitor, KB-R7785, in two tumor models. Cancer Res. 59, 1252–8 (1999). 10096556

[b59] Blezinger P. *et al.* Systemic inhibition of tumor growth and tumor metastases by intramuscular administration of the endostatin gene. Nat Biotechnol. 17, 343–8 (1999). 10207881 10.1038/7895

[b60] Niethammer A. G. *et al.* A DNA vaccine against VEGF receptor 2 prevents effective angiogenesis and inhibits tumor growth. Nat Med. 8, 1369–75 (2002). 12415261 10.1038/nm1202-794

[b61] Chou T. C. Theoretical basis, experimental design, and computerized simulation of synergism and antagonism in drug combination studies. Pharmacol Rev 58, 621–81 (2006). 16968952 10.1124/pr.58.3.10

